# A Cross-Correlational Analysis between Electroencephalographic and End-Tidal Carbon Dioxide Signals: Methodological Issues in the Presence of Missing Data and Real Data Results

**DOI:** 10.3390/s16111828

**Published:** 2016-10-31

**Authors:** Maria Sole Morelli, Alberto Giannoni, Claudio Passino, Luigi Landini, Michele Emdin, Nicola Vanello

**Affiliations:** 1 Institute of Life Science, Scuola Superiore Sant’Anna, 56127 Pisa, Italy; mar.morelli@sssup.it (M.S.M.); passino@ftgm.it (C.P.); emdin@ftgm.it (M.E.); 2 Research Center “E. Piaggio”, University of Pisa, 56122 Pisa, Italy; 3 Fondazione Toscana Gabriele Monasterio, National Research Council, 56124 Pisa, Italy; alberto.giannoni@ftgm.it (A.G.); luigi.landini@iet.unipi.it (L.L.); 4 Dipartimento di Ingegneria dell’Informazione, University of Pisa, 56124 Pisa, Italy

**Keywords:** EEG, missing data segments, cross correlation function, control of breathing

## Abstract

Electroencephalographic (EEG) irreducible artifacts are common and the removal of corrupted segments from the analysis may be required. The present study aims at exploring the effects of different EEG Missing Data Segment (MDS) distributions on cross-correlation analysis, involving EEG and physiological signals. The reliability of cross-correlation analysis both at single subject and at group level as a function of missing data statistics was evaluated using dedicated simulations. Moreover, a Bayesian-based approach for combining the single subject results at group level by considering each subject’s reliability was introduced. Starting from the above considerations, the cross-correlation function between EEG Global Field Power (GFP) in delta band and end-tidal CO_2_ (P_ET_CO_2_) during rest and voluntary breath-hold was evaluated in six healthy subjects. The analysis of simulated data results at single subject level revealed a worsening of precision and accuracy in the cross-correlation analysis in the presence of MDS. At the group level, a large improvement in the results’ reliability with respect to single subject analysis was observed. The proposed Bayesian approach showed a slight improvement with respect to simple average results. Real data results were discussed in light of the simulated data tests and of the current physiological findings.

## 1. Introduction

An electroencephalogram (EEG) can record electrical brain oscillations from electrodes on the scalp surface and can be analyzed through different signal processing approaches, such as time domain, frequency domain, and time-frequency analysis methods [1,2]. Although the analysis of EEG signals has a long history within the neuroscience field, the reciprocal influence among EEG signals and other physiological processes, such as heart regulatory processes or breathing control, is still to be fully characterized [3,4]. One approach that allows for merging EEG-related information and specific physiological signals is represented by the use of the cross-correlation function in the time domain [5]. This method identifies the degree of similarity between two signals and is largely used to evaluate brain connectivity [6,7]. In [8], it was used to estimate the time delay between drug delivery and bispectral index used to control anesthesia level in a closed loop model for an Intensive Care Unit. The analysis of cross-correlation function was adopted to evaluate the link between EEG and cerebral blood flow in [9] to improve the knowledge of cerebral blood flow control in newborns. In [10], a correlational analysis among respiratory volume per time, EEG, and Blood Oxygen Level Dependent (BOLD) signals was performed using concurrent EEG-fMRI acquisition at rest. The correlational analysis results allowed us to hypothesize a reciprocal link of neuronal origin among EEG power, respiration, and BOLD signal. 

One relevant methodological issue in the analysis of the EEG signal is the presence of several kinds of artifacts [11] that might cause a loss of information [12], a decrease of Signal-to-Noise Ratio (SNR), and an alteration in the results of the cross-correlation analysis [9]. While in Event-Related Potential (ERP) studies SNR is improved by averaging several trials that are time-locked to a stimulus [13], this approach cannot be followed in cross-correlation analysis. Artifacts showing high reproducibility can be effectively removed by using multivariate approaches such as Principal and Independent Component Analysis (PCA and ICA) [14,15]. For instance, in [16], an automatic method was developed to identify eye blink-related component. Specifically, this approach is based on a measure of correlation between the independent components and frontal EEG signals, together with the analysis of the spatial distribution of the power of the component. In another work [17], a semi-automatic cleaning method based on ICA and the definition of statistical markers to classify the components, was proposed to improve the quality of the EEG signal acquired from Alzheimer’s patients. However, in the case of non-stationary phenomena, electrodes, or body movements, artifact removal cannot be easily accomplished, and corrupted data segments must be discarded. As a matter of fact, the presence of Missing Data Segments (MDS) is a common problem in the analysis of EEG signals that can influence the validity and the interpretability of research results [18]. A method that allows us to estimate the cross-correlation function in the presence of MDS was developed in [9]. In the above-cited work, an approach based on the estimation of the cross-correlation function between incomplete EEG data and cerebral blood flow was presented. Moreover, Monte Carlo simulations were carried out to estimate the distribution of the cross-correlation function under the null hypothesis of no correlation (H_0_), thus assessing the statistical significance of the observed results. The statistical power of the analysis was also evaluated using exactly the same missing samples observed in each subject. Some tests were also performed by randomizing the same number of missing samples found in real acquisitions. The above-cited work has one limitation. In fact, it does not take into account the effect of different distributions of MDS on the correlation function between the unknown, noise-free signals. In fact, different experiments are likely to differ as to number and length of MDS. In [19], Morelli et al. partially addressed this problem by evaluating the effects of a limited number of MDS distributions on a correlational analysis between simulated EEG Global Field Power (GFP) and End tidal CO_2_ (P_ET_CO_2_). 

Here, we aim at expanding the above-cited work to quantify the validity and reliability of the results in the presence of MDS. This goal will be pursued by carrying out extensive simulations to mimic the impact of different MDS distributions on the correlation analysis between GFP and P_ET_CO_2_ signals. The simulations will be performed both at single subject and at group levels.

Moreover, a novel approach to performing a group analysis in the presence of MDS will be presented. The proposed approach aims at estimating a measure of reliability of the cross-correlation function between EEG power and P_ET_CO_2_ at the single subject level, and adopting this measure to correct the analysis at the group level within a Bayesian framework. 

The choice of performing the correlation analysis between EEG and blood-related CO_2_ changes is motivated by the need to improve current knowledge in the central processes involved in the control of breathing. Specifically, such an investigation could reveal how cortical activations are involved in the regulation of breathing both in physiological and pathological conditions, such as central apneas, Cheyne-Stokes Respiration (CSR) [20], and Obstructive Sleep Apnea (OSA) [21]. In this work we will focus on the brain activity in the delta band, which was found to be correlated with CO_2_ level changes in healthy subjects [22]. Moreover, significant changes of EEG activity in the delta band EEG were observed in OSA [23] and in CSR subjects [24]. In view of the methodological findings and available literature on this topic, a correlational analysis between EEG signals and P_ET_CO_2_ signals recorded during Free Breathing (FB) and Breath Hold (BH) tasks in healthy subjects will be performed and discussed.

## 2. Materials and Methods

### 2.1. Experimental Protocol

Six healthy subjects (all males, age 31 ± 7) were recruited for this study. An EEG device and a system for physiological parameters acquisition were used for simultaneous acquisition of brain activity and physiological parameters related to breathing and heart functions. The 64-electrode EEG device (Compumedics USA, Charlotte, NC, USA) included two channels for the Electrocardiographic (ECG) signal and two channels for Electro-oculogram (EOG) signal. The EEG signal was low-pass filtered at 400 Hz and sampled at 1 kHz. The electrodes’ impedance was kept below 30 kΩ during all recordings. Exhaled CO_2_ and blood oxygen saturation (SpO_2_) were recorded with a CO_2_ analyzer (Novametrix Medical Systems Inc., Wallingford, CT, USA) and with a pulse oximeter (Minolta, Tokyo, Japan), respectively. The physiological signals were digitized online via a National Instrument acquisition card and a specific homemade software (Fondazione Toscana Gabriele Monasterio, National Research Council, Pisa, Italy) written in Java. The subjects performed two different tasks. In the FB task the subject had to breathe normally while lying down with eyes closed for 360 s. The second one was a voluntary end-inspiratory BH task: the subject, after 1 min of FB task, was asked to alternate 5 cycles of 30 s of BH and 30 s of FB, for a total length of 360 s. Subjects were advised to start and stop the BH task by touching their left leg. The same touching procedure was adopted in the FB task, for control purposes. The experimental protocol was approved by the Institutional Ethical Committee. 

### 2.2. EEG Processing

The recorded EEG data were filtered with a Hann pass filter between 1 and 30 Hz. Bad electrodes were detected for each acquisition and removed from the analysis. Blink and pulse artifacts were detected using EOG and ECG signals and removed using PCA algorithm. All data segments in which artifact removal methods were not effective were marked and excluded from subsequent analysis. These segments were defined as Missing Data Segments (MDS). A time-frequency analysis was performed to estimate the EEG power in the delta (1–4 Hz) band in the remaining Valid Signal segments (VS). For each channel, the spectrogram was calculated using a Hanning window of 2 s length and overlapping time of 1 s. The GFP in delta band was calculated as the mean power value across all channels. Finally, the calculated GFP was resampled at 50 Hz and linearly detrended. 

### 2.3. Physiological Signal Processing

The observation of SpO_2_ levels was used to detect the tasks’ possible effects on oxygen level. The normal range of SpO_2_ was considered to lie between 95% and 100%. The exhaled CO_2_ waveforms were used to estimate the P_ET_CO_2_ time series, which is an estimate of arterial CO_2_ (P_a_CO_2_) [25]. In both tasks, during the breathing period, the CO_2_ values at the end of each expiration phase were interpolated to calculate P_ET_CO_2_. In the BH task, during BH intervals the P_ET_CO_2_ signal was estimated using a cubic spline model interpolation. As for GFP, the P_ET_CO_2_ signal was linearly detrended and resampled at 50 Hz to facilitate the alignment of both time series.

### 2.4. Single Subject Correlational Analysis

In this section, the methodology used to estimate the Cross-Correlation Function (CCF) between GFP and P_ET_CO_2_ at single subject level will be described. First, it will be shown how the CCF can be evaluated in the presence of MDS (Section 2.4.1). Then, the approach used to quantify the impact of MDS on the CCF estimate, using simulated data, will be detailed (Section 2.4.2). The results of the latter study will be used both to evaluate the reliability of the single subject analysis results and to inform the group level analysis, as will be described in Section 2.5. Finally, a description of the analysis of real data at single subject level will be given (Section 2.4.3). In this case, an approach using surrogate data will be followed to evaluate the statistical significance of the results.

#### 2.4.1. CCF Estimation

The CCF between GFP and P_ET_CO_2_ will be estimated in MATLAB (MATLAB, The Mathworks Inc., Natick, MA, USA) for time lags between −30 s and 30 s. When MDS are present, the CCF is estimated taking into account only EEG VS segments after the exclusion of MDS. Specifically, the correlation coefficient at each time lag was obtained using only the intersection between the VS of the GFP and P_ET_CO_2_ time series. 

#### 2.4.2. Evaluation of the Effects of MDS on Correlational Analysis

We propose to evaluate the effects of MDS on the correlation analysis using dedicated simulations. We hypothesize that the estimate of the CCF between EEG-GFP and P_ET_CO_2_ depends upon the length and the time distribution of MDS as well as upon different possible time courses of EEG-GFP and P_ET_CO_2_. We hypothesize that the distribution of MDS is the realization of a random process that is independent from the process generating the EEG-GFP signal. Several MDS distributions, differing for total length and number of segments, were simulated. Each MDS was applied to simulated MDS-free GFPs in the delta band and the corresponding CCF were estimated. The approach we followed is characterized by the following steps:

(i) *MDS simulations*. Five classes of MDS, characterized by increasing percentage of VS, were simulated (class A: 50%–60% of VS, class B: 60%–70% of VS, class C: 70%–80% of VS, class D: 80%–90% of VS, class E: 90%–100% of VS). For each class, 30 different MDS distributions were extracted. The constraints on MSD length were obtained from real data.
(ii) *GFP simulations*. The MDS-free GFP time courses were simulated to have the same second order statistics (i.e., power spectrum) of the measured GFPs in the delta band. Since observed GFPs usually contain MDSs, to obtain MDS-free GFPs we choose to adopt the approach developed in [9]. Specifically, the GFP surrogate time series was obtained from the AutoCorrelation Function (ACF) estimated exploiting available data, under the hypothesis that the ACF obtained with and without MDS are not significantly different. The GFP amplitude spectrum was given by the square root of the Fourier Transform (FT) of the ACF. Then a random phase was added to the GFP amplitude spectrum. Finally, a MDS-free surrogate GFP time series was obtained by applying the Inverse FT to the GFP spectrum (see Appendix A). Among surrogate data, we chose the GFP time series that resulted in the cross-correlation with P_ET_CO_2_ having the maximum value at time delay equal to 0 s. We chose to simulate three GFP families. Specifically, we simulated 10 GFPs with a maximum, and statistically significant, correlation with P_ET_CO_2_ equal to 0.3 ± 0.05, 10 with a maximum correlation coefficient equal to 0.4 ± 0.05 and 10 GFPs with a maximum correlation of 0.5 ± 0.05.
(iii) *Final evaluation of the effects of MDS on CCF estimation*. The CCF time courses, the maximum correlation coefficients (close to 0.3, 0.4, and 0.5, as described in the previous paragraph), and the corresponding time lags (corresponding to t = 0 s), which were obtained from MDS-free GFP time series, represent the “true” values, referred to in the following as *target values*. The CCF and corresponding statistics obtained by applying the MDS to the simulated GFPs will be referred to as *actual values*. A total of 300 different CCFs for each combination of MDS class and GFP family were obtained. Different quality parameters were evaluated. First, the differences between the amplitudes of the target and actual maximum correlation coefficients as well as the differences in the corresponding time lags were estimated. Then, the differences between actual and target CCF time series were quantified by estimating the Normalized Root-Mean-Square Error (NRMSE) that can be seen as a measure of accuracy (see Appendix B). Finally, the standard deviations of the CCFs at each time lag were calculated, thus obtaining a measure of the precision as a function of the VS percentage and GFP family.

All the simulations were performed in Matlab using a desktop computer (Intel (R) Core (TM) i7-3700 CPU @ 3.50 GHz, RAM 8 GB, operating system Windows 7–64 bit). A time interval of 30 min was necessary to estimate the 10 simulated GFPs in each group of target values. For each combination of simulated GFP and MDS distribution, the calculation of CCF required approximately 100 s. A parallel computing approach was adopted using the *parpool* Matlab function. Specifically, the *parfor* function was used to execute a *for* loop in parallel using four workers. 

#### 2.4.3. Analysis of Real Data at Single Subject Level

Since the phenomena of interest are characterized by slowly varying components, the GFP and P_ET_CO_2_ time courses were smoothed with a zero-phase moving average filter of 10 s before correlation analysis [10]. At each time lag the value of the CCF, i.e., the cross-correlation coefficient, can be seen as an observed value of a random variable that depends on the actual GFP and P_ET_CO_2_ time courses. The statistical significance of the results was assessed by estimating the distribution of the CCF under the null hypothesis of no correlation between signals (H_0_) using surrogate data. To achieve this goal, non-parametric method described in [26] was used. Specifically, the Fourier Transform (FT) of the original signals was performed and 1000 phase randomized surrogates were generated for each signal and for each time lag. When MDS were observed in the GFP signal, the GFP surrogate time series was obtained from the ACF estimated exploiting available data as described in Section 2.4.2. (ii). As a result, 1000 estimates of the CCF under H_0_ were obtained. The critical values of the correlation coefficients, corresponding to α = 0.05, were estimated from percentile values as a function of time lag. The computing time to estimate each CCF was approximately 100 s, as described in Section 2.4.2.

### 2.5. Group Analysis

At the group level we propose to adopt a Bayesian approach whereby the information about the proportion between VS and MDS is exploited. A classical approach to estimate the CCF at group level is to average the functions obtained at the single subject level. Our aim is to improve the simple average approach by weighting the contribution of each subject to the group level CCF. The weights are related to a reliability measure estimated from the simulated data at the single subject level. We propose using the inverse of the CCF standard deviation estimated, as described in Section 2.4.2. (iii). Specifically, a standard deviation value at each time lag was estimated for each one of the five MDS classes, by averaging all the values obtained across the GFP families. Our approach is derived from the group level analysis described in [27]. This method consists of combining results from single subject analysis in an iterative way, starting from the probability distribution of the parameter of interest. According to the Bayes rule (see Appendix C), it is possible to write the posterior distribution of the parameter, given the results from the first subject $y_{1}$, as

(1) $$p\left(\theta |y_{1}\right)\propto l\left(\theta |y_{1}\right)p\left(\theta \right),$$

where $l\left(\theta |y_{1}\right)$ is the likelihood of *θ* given the data and p(θ) is the prior about the parameter [28]. In this study a flat prior was adopted. The iterative scheme consists in treating the posterior distribution after the first subject acquisition in Equation (1) as a prior for the second observation $y_{2}$, whose posterior distribution can be written as

(2) $$p\left(\theta |y_{1},y_{2}\right)\propto l\left(\theta |y_{2}\right)l\left(\theta |y_{1}\right)p\left(\theta \right)=l\left(\theta |y_{2}\right)p\left(\theta |y_{1}\right).$$

This scheme will be repeated for n subjects and under the hypothesis of Gaussian likelihood functions, the mean parameter at group level will have posterior Gaussian distribution $N\left(\bar{\theta },{\bar{\sigma }}^{2}\right)$, characterized by [27,28].

(3) $$\begin{array}{ccc} \bar{\theta }=\frac{\sum_{i=1}^{n}\sigma_{i}^{-2}\theta_{i}}{\sum_{i=1}^{n}\sigma_{i}^{-2}} & & {\bar{\sigma }}^{2}=\frac{1}{\sum_{i=1}^{n}\sigma_{i}^{-2}} \end{array}$$

Equation (3) is a weighted average where each parameter’s variability, σ_i_, can be seen as a measure of the reliability for each subject. Using such an approach, each subject is considered to contribute with a weight that is inversely proportional to her/his corresponding reliability measure. In this study, the parameter of interest $\bar{\theta }$ is the correlation coefficient at each time lag at the group level. We can explicitly indicate its time dependency as $\bar{\theta }\left(\tau \right)$. To estimate this parameter we consider $\theta_{i}\left(\tau \right)$ as the value of the CCF of the *i*-th subject at time lag τ. The variability of the parameter $\theta_{i}\left(\tau \right)$ can be written as $\sigma_{i}\left(\tau \right)$ and is related to the standard deviation of the CCF obtained using simulated data, as in Section 2.4, and chosen according to the amount of VS observed in the *i*-th subject recordings. In other words:

(i) Each subject *i* is assigned to a class according to the percentage of VS with respect to the overall signal length. Five classes are considered, as described in Section 2.4 (class A: 50%–60% of VS, class B: 60%–70% of VS, class C: 70%–80% of VS, class D: 80%–90% of VS, class E: 90%–100% of VS);
(ii) The variability of the CCF for the *i*-th subject, at each time lag, is derived from the distribution of the correlation coefficients found from the simulations described in Section 2.4;
(iii) At each time point, to calculate the correlation coefficient at group level, each subject is weighted considering its class.

#### 2.5.1. Test on Simulated Datasets 

The effectiveness of the proposed approach was first tested on simulated datasets with known cross-correlation functions. Specifically, each subject in the group had the same GFP time course but a different MDS distribution. The target cross-correlation function at the group level was the average cross-correlation function obtained without MDS. A comparison of the CCFs obtained using the simple average and the weighted average methods was performed. The effectiveness of both approaches was evaluated by calculating the NRMSE between the target and the actual cross-correlation functions. Moreover, the amplitude and the corresponding time shift of the maximum correlation coefficients were taken into consideration. Four different tests, differing by target CCFs as well as MDS distributions, were performed:

(i) Fifteen subjects were taken into account. The GFP and P_ET_CO_2_ were simulated to obtain a CCF with a maximum correlation coefficient equal to 0.5 at a zero time lag. The five MDS classes were equally distributed among the subjects (20% of subjects belonged to class A, 20% of subjects belonged to class B, 20% to class C, 20% to class D, and 20% to class E).
(ii) Fifteen subjects were taken into account. The GFP and P_ET_CO_2_ were simulated to obtain a CCF with a maximum correlation coefficient equal to 0.5 at a zero time lag. A different distribution of MDS with respect the preceding point was applied (40% of subject to class A, 40% to class B, and 20% to class E).
(iii) The same distribution of MDS as in point (ii) was also tested on a GFP whose target maximum correlation with the P_ET_CO_2_ was equal to 0.7 at a zero time lag.
(iv) The CCF is tested using the same number of subjects (i.e., six subjects) we enrolled in the real data study and the same MDS distributions we experimentally observed. Both GFP and P_ET_CO_2_ time series were simulated to have a maximum correlation coefficient equal to 0.5 ± 0.05 at a zero time lag.

#### 2.5.2. Analysis of Real Datasets 

The average and the weighted average CCFs were estimated on the group of subjects under study. For the weighted average approach, each subject within the group was associated with an MDS distribution according to the percentage of VS (class A, B, C, D, and E, as described above). The final weighted average CCF was compared to the group averaged CCF. The statistical significance of each curve was obtained using surrogate data. Specifically, 1000 surrogates were estimated for each time lag and the threshold values corresponding to the 0.05 significance level were approximated from percentile values.

## 3. Results

### 3.1. MDS Statistics

In Table 1, the statistics related to the simulated MDS are shown. Different percentages of VS, number and length of missing data segments were obtained starting from the MDS statistics observed in real datasets.

### 3.2. Single Subject Analysis 

#### 3.2.1. Simulated Data 

In Table 2 the correlational analysis results (time delay and cross-correlation coefficient) using different families of simulated GFPs and different MDS distributions are shown. 

**Figure 1:**
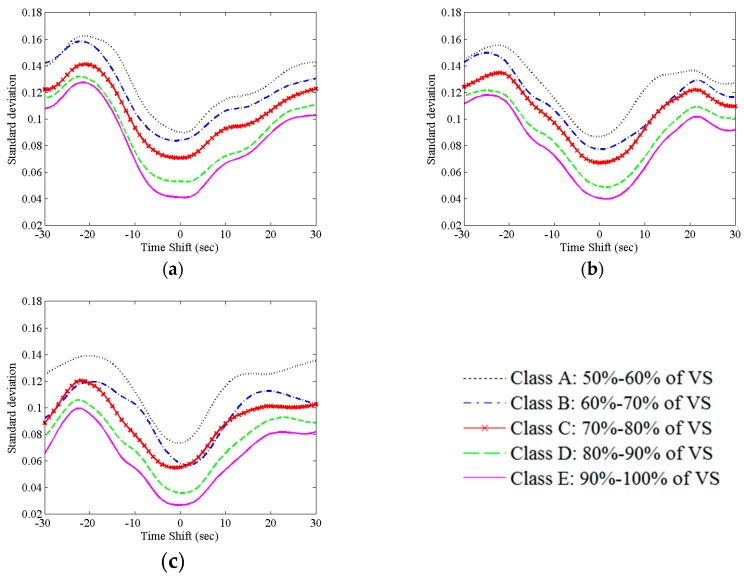
Standard deviation values of cross correlation function in simulated data for different GFPs family ((**a**) Maximum CCF = 0.3 ± 0.05; (**b**) Maximum CCF = 0.4 ± 0.05; (**c**) Maximum CCF = 0.5 ± 0.05) and MDS classes. In each family, with a decrease in VS percentage there is an increase in standard deviation, that ism a loss of precision in simulations. Comparing the three panels, it is possible to observe that precision improves in classes with an increased maximum correlation coefficient.

A reduction of the expected maximum correlation coefficient as well as an increase of its standard deviation is obtained with decreasing percentages of VS. The decrease of expected maximum correlation coefficient and the increase of standard deviation highlight a severe accuracy and precision loss, respectively. Both accuracy and precision (Figure 1) improve with increasing values of simulated maximum correlation coefficient.

**Figure 2:**
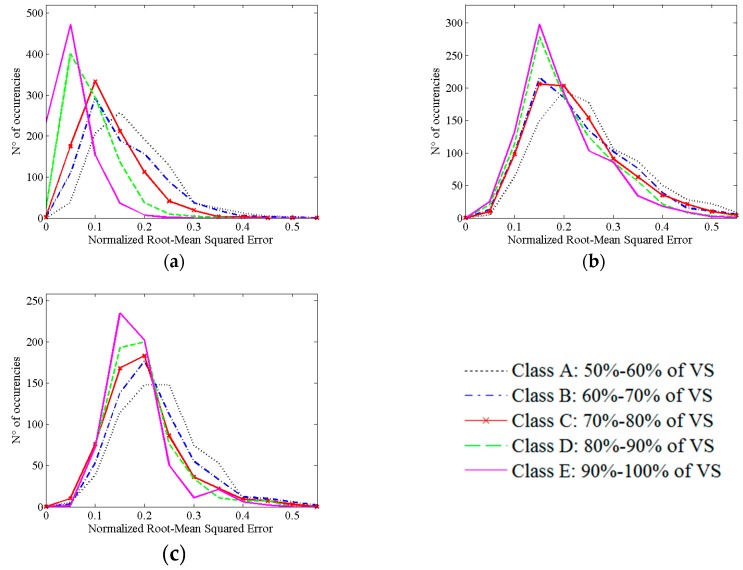
Distribution of Normalized Root Mean Squared Error (NRMSE) between estimated and theoretical cross correlation functions at single subject level. The figures are related to cross correlation functions with theoretical maximum correlation coefficient equal to 0.3 (**a**); 0.4 (**b**); and 0.5 (**c**). The different lines are related to different groups of MSD distributions.

Accordingly, the time delays corresponding to the maximum correlation coefficient show large departures from theoretical values. We noticed how large values can also be found with large percentages of VS. Moreover, in Table 2 the mean value of standard deviation of the simulation across all time lags is shown. Also in this case, a loss of precision is observed along with the decrease of VS percentage. To evaluate the accuracy, NRMSE was studied (Figure 2). A reduction of NRMSE error (see Appendix B) with increasing percentages of VS was found. 

#### 3.2.2. Real Data Results 

**Figure 3:**
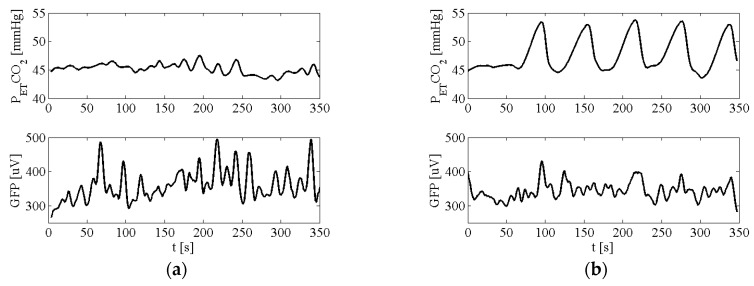
P_ET_CO_2_ and GFP signals of Subject 4 recorded during FB task (**a**) and BH task (**b**).

In Figure 3, the time courses of P_ET_CO_2_ and GFP signals during both breathing tasks for one subject are shown as an example.

During both tasks, besides EEG and CO_2_ signals, an SpO_2_ signal was recorded. No significant changes were observed in SpO_2_ during the FB and BH tasks. The observed maximum variations of CO_2_ during free breathing across all subjects was 4.9 ± 2.1 mmHg, while during the breath holding task it was 10.7 ± 1.3 mmHg. The average number of discarded electrodes across all subjects was three, with a maximum of 7 in one acquisition. The spatial distribution of the removed electrodes, in all the cases, was sparse.

For each subject and each task, the statistics of missing samples were evaluated. The number and length of missing segments, the percentage of removed signal, and the percentage of valid signal are reported in Table 3.

In Table 4 the results of correlational analysis applied to each subject are reported including the time shift for the maximum correlation, the maximum correlation coefficient, and the critical values for the significance of the correlation coefficient.

The maximum correlation values for positive time shifts were observed during the BH task, in almost all cases. The maximum correlation coefficient was positive in all subjects except two. Specifically, in one subject a positive correlation coefficient was found, but the observed value was slightly below the critical value. In another subject, a negative correlation was found. In the FB task, although significant results were found in most of the subjects, the observed results are less coherent with respect to those obtained in the BH task. Specifically, the signs of the time shifts and the values of the maximum correlation coefficients were less homogeneous across the subjects in the FB task with respect to the BH task.

### 3.3. Group Analysis

#### 3.3.1. Simulated Datasets Results 

**Figure 4:**
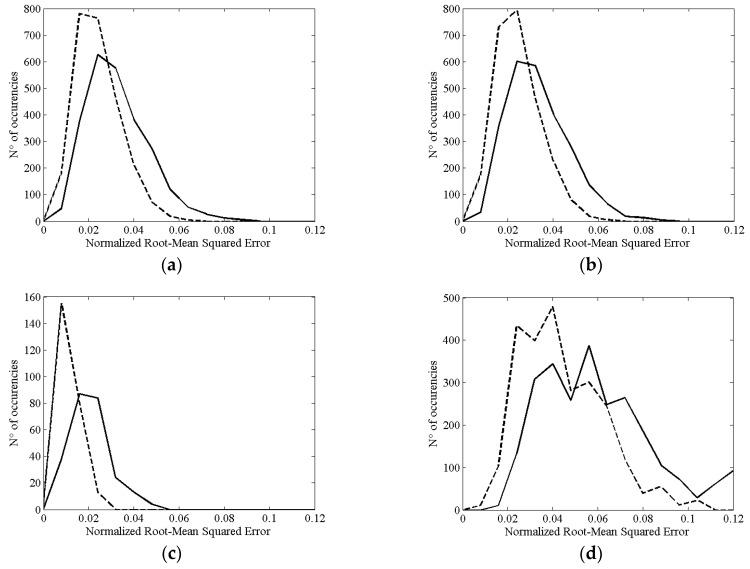
Distribution of Normalized Root Mean Squared Error between estimated and theoretical cross-correlation functions. Two curves are shown for each case: the difference with the weighted average approach (dotted line) and the difference with the simple average (solid line). (**a**) Equal distribution of missing data segments groups across subjects; (**b**) 40% group A, 40% group B, and 20% group E (see Table 1); (**c**) Results related to the GFP with maximum correlation coefficient equal to 0.7; 40% group A, 40% group B, and 20% group E; (**d**) Simulations with six subjects mimicking the missing data segments distribution observed in our study.

The analysis of simulated datasets showed that the weighted average approach allows us to improve the estimation of the cross-correlation function with respect to the simple average. In Figure 4, the histograms of the NRMSE calculated between the two estimates, weighted average and simple average, and the theoretical cross-correlation function are shown for the four simulated scenarios.

In the first case (Figure 4a), 15 subjects were chosen to have a homogeneous distribution of MDS distributions across all groups. In a second case (Figure 4b), 40% of the subjects were chosen to have a VS percentage falling into the 50%–60% range, another 40% belonged to the 60%–70% range, while the rest had a VS percentage equal to 90%–100%. While the NRMSE is low in both cases, the weighted average showed better results with respect to the simple average approach. The results related to the simulations using GFP time courses with a maximum correlation coefficient equal to 0.7 are shown in Figure 4c. Even in this case an improvement of NRMSE was achieved using the weighted average approach. A simulation was also performed mimicking the characteristics of our study in terms of the MDS distributions and the number of subjects (Figure 4d). The maximum correlation coefficients along with the corresponding time shifts for the group level analysis are shown in Table 5. Weighted and simple average results in this case are very similar. Only a small improvement regarding time delay estimation was achieved using weighted average.

#### 3.3.2. Real Dataset Results 

**Figure 5:**
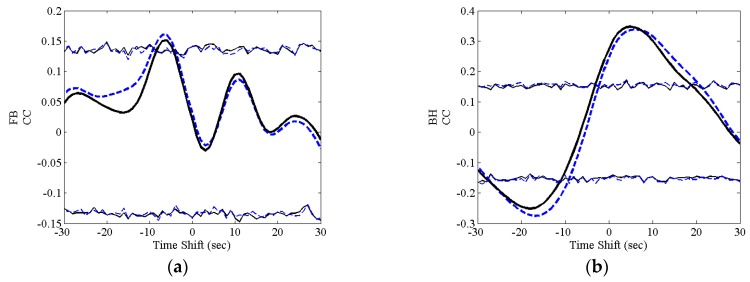
Time courses of the cross-correlation functions as a function of the time shift. Dashed blue lines represent the simple average while the solid black line is the weighted average. The thin lines represent the level of significance for simple average (thin dashed blue line) and weighted average (thin solid line). (**a**) FB related results; (**b**) BH results.

In Figure 5, the time courses of the cross-correlation functions estimated at group level using both the weighted and simple average for delta band are shown. 

The statistical thresholds that correspond to a significance level for the null hypothesis of no correlation equal to α = 0.05 are also shown. The results for weighted average and simple average are very similar, as can be seen by looking at Figure 5. Some differences in the time courses obtained using the two approaches were observed. However, very small differences in the results were reported regarding the maximum correlation coefficients and the corresponding time shifts. In Table 6 the maximum correlation coefficients as well as the corresponding time shifts are shown. Specifically, slightly different time shifts were obtained by the two approaches. 

Positive correlation coefficients were found. This implies that the changes of GFP in the delta band are coherent with the changes in P_ET_CO_2_. A positive time shift for maximum correlation was found for BH task, while the maximum correlation was found for a negative time shift in the FB task. A positive time shift implies that the GFP changes follow the P_ET_CO_2_ changes. 

## 4. Discussion

In this study, we aim at evaluating the effects of MDS on cross-correlation analysis both at the single subject and the group level. MDS represents a relevant issue in EEG data analysis since some categories of EEG artifacts cannot be easily reduced and might imply a data rejection step. As a result, a negative impact of MDS on correlational analysis might be significant. Simulations were thus carried out to estimate the variability on the estimated CCF, related to different possible distributions of MDS in the EEG recordings. We have to stress that we made some choices about the possible GFP time courses where we applied the MDS distributions. Specifically, we hypothesized a maximum correlation coefficient ranging from 0.3 to 0.5. These correlation coefficients, given the number of time points in the series, are likely to correspond to significant values. In fact, we were interested in evaluating the sensitivity of the approach by looking at false negative events, i.e., evaluating the probability of accepting a false null hypothesis of uncorrelated signal (type II errors). In principle, it would also be possible to analyze the effects of MDS on GFPs, showing a lower correlation coefficient with a given P_ET_CO_2_ time course. In simulation analysis, at the single subject level, our findings suggest that even a small percentage of MDS can considerably modify the correlational analysis results. Specifically, the time shift for the maximum correlation coefficient showed a large departure from the expected level. In particular, both the precision and the accuracy of the maximum correlation coefficient and CCF estimates were worse with increasing percentages of missing data segments. Regarding group-level analysis, the averaging of the cross-correlation functions allowed us to improve the reliability of the results. We have to stress that these results were obtained starting from a hypothesis of significant coherent correlation across subjects. This means that we also focused on type II errors for the group-level study. 

To our knowledge, this study introduces one methodological novelty for group level analysis results. Specifically, we propose estimating the variability at each time point of the cross-correlation function due to missing data segments distribution and using it to weight the contribution of each subject to the group level statistics. The proposed solution was developed within a Bayesian framework since the reliability of the *i*-th subject measure is based on a model of the data generating process, and not on the actual measured data. This method was found to outperform the simple average approach, as can be seen by the analysis of the NRMSE between estimated and theoretical cross-correlation functions. The advantages evaluated by the proposed simulations in terms of maximum correlation coefficient and corresponding time shifts’ precision were not significant. The reliability measures, estimated for a small range of possible GFPs time courses, would not allow us to generalize the results of the proposed approach. To partially address this issue, a simulation was carried out using a GFP that showed a maximum correlation with coefficient P_ET_CO_2_ equal to 0.7, thus outside of the range used for estimating the reliability measure. The weighted average method even in this case was shown to improve the results at the group level with respect to those obtained with the simple average. This result suggests a robustness of the proposed approach with respect to the effect size misspecification, i.e., the unknown degree of correlation between the two signals’ time courses. Obviously, the achievable improvement with respect to simple average approach is related to the simultaneous presence of good and bad acquisitions within the same group study. Future investigations could explore the impact of MDS on the correlational analysis as well as quantify the benefits of the proposed Bayesian-based correction at the group level in other EEG frequency bands. 

In light of the above considerations, the cross-correlation function between GFP in delta band and P_ET_CO_2_ were calculated during both FB and BH tasks from data acquired from healthy subjects. GFP is a measure of global electrical activity, and it is quite robust with respect to electrode choice or distribution [29]. For this reason, the number of discarded channels we obtained is acceptable for this study. The analysis of the correlation between EEG and variation in CO_2_ level could represent a useful tool to understand how cortical activations are involved in the control of breathing both in physiological and pathological conditions. In particular, the BH task represents a maneuver to observe the cortical effect due to variations in P_ET_CO_2_ with generation of CO_2_ wave changes, better resembling pathological conditions such as central apneas and OSA. Actually, considering these particular patient populations, the comprehension of control of breathing could have a great impact. Different methods use EEG to observe the effects of apnea in patients [23,24,30,31,32,33], and although different kinds of treatment were proposed both to OSA [34] and CSR, these pathologies have a great impact on patients’ quality of life. Indeed, untreated patients with OSA have increased risk of cardiovascular events, death during sleep, and stroke. On the other hand, CSR is common in heart failure (HF) patients (30%–70%) and has a predictive value for morbidity and mortality in HF [35]. Regarding the study on healthy subjects here presented, we have to stress that the interpretation of these results is limited by the small number of subjects recruited. However, the results show significant and coherent trends across subjects. Moreover, considering the observed percentage of MDS in each subject and the simulated tests results, we expect the analysis at group level to be more robust against the presence of MDS than single-level analysis. Given the above considerations, although future studies are needed involving a larger number of healthy subjects and patients, some hypotheses can be formulated. At the group level, in delta band it is possible to highlight that a positive maximum correlation coefficient was found in both tasks but the corresponding time shifts moved from a negative to a positive value passing from FB to BH tasks. This means that during FB variations of the GFP signal precede the P_ET_CO_2_ changes, while the opposite occurs during the BH task. A possible mechanism explaining the difference between the two tasks in our results is that during FB, the -Bötzinger complex in the brainstem controls respiratory motor neurons, respiratory muscles, and pulmonary ventilation [36]. At rest and especially in the awake subject, the relative weight of the chemical control of ventilation driven by small variations in CO_2_ might have a lower impact on breathing activity, and therefore on ventilation-related cortical activity. It is likely that variation in the respiratory pacemaker centers, influenced by different triggers, may randomly change ventilation, with an effect on plant gain (the lung), and therefore on CO_2_ levels. On the contrary, during the BH task the progressive increase in CO_2_ (on average around 10 mmHg of CO_2_ increase) may actually change the pre-Bötzinger discharge and therefore the cortical activity linked to ventilation. Also, in studies that use hypercapnic stimuli, an increase in the delta band was found to follow an increase in CO_2_ levels, in all electrodes across the whole brain [22]. The increase in the delta band was in accordance with the positive correlation between CO_2_ and GFP found in our study. Our results have some similarities with those obtained in [23], where Thomas studied OSA patients. Specifically, at the end of the apnea, characterized by higher CO_2_ values, an increase in delta power was observed. On the contrary, in patients with heart failure suffering from central apneas/Cheyne–Stokes respiration, an increase in the delta power band was observed with some delay, in the first part of the hyperpnea phase [24]. This phenomenon may be due to a different contribution of hypoxia or to the circulatory delay between the alveolar level and the chemoreceptors typical of the disease.

## 5. Conclusions

Missing segments in EEG signal may have a severe impact, especially in correlation analysis between EEG and non-stationary signals such as CO_2_ and ventilation. This study proposes a way to evaluate the influence of MDS on cross-correlational analysis, specifically in the relationship between EEG and P_ET_CO_2_ in simulated EEG data and in real signals recorded in healthy subjects. The present findings based on simulated data show that MDS have a significant impact. Specifically, even a small amount of MDS may considerably reduce the reliability of the results. Group-level analyses were also evaluated. This study shows that, under the hypothesis of a significant correlation between the two signals, the group-level analysis is more robust with respect to MDS statistics. Moreover, a Bayesian-based approach was proposed to correct the analysis at group level, taking into account a reliability measure estimated at the single subject level and related to the amount of missing data. The proposed approach allowed us to achieve an improvement in the final results.

Considering the real data, in healthy subjects undergoing contemporary monitoring of EEG and P_ET_CO_2_ the different behavior of the two signals may be observed during the FB and BH tasks. Interestingly, in the delta band, a specular time shift is commonly observed at group level during the two tasks. Considering the complexity of the physiological and pathophysiological mechanisms linking CO_2_ to cerebral activity, the precision and accuracy of signal analysis may help us avoid misinterpretation of study results. 

## Figures and Tables

**Table 1:** Statistics of simulated Missing Data Segments (MDS) and Valid Signal (VS) percentages.

Class Label	Percentage of VS	VS Statistic (s)		Number of MDS (mean)	MDS Statistics (s)	
		Mean Length	Standard Deviation Length		Mean Length	Standard Deviation Length
A	50%–60%	14.00	5.9	13	12.01	2.8
B	60%–70%	21.27	10.4	10	12.04	3.1
C	70%–80%	32.22	17.5	7	11.68	2.9
D	80%–90%	56.70	33.8	4	11.70	2.2
E	90%–100%	142.41	31.8	2	12.97	2.0

**Table 2:** Correlational analysis for simulated data with different MDS distribution. (**A**) Results for time delay of maximum correlation coefficient; (**B**) Results for maximum correlation coefficient; (**C**) Cross-correlation function (CCF) precision (mean value of standard deviation of CCF with respect to target value, for each time shift). The results are shown for different target maximum correlation coefficients (T. Max.ρ).

T. Max. ρ	A (50%–60% of VS)	B (60%–70% of VS)	C (70%–80% of VS)	D (80%–90% of VS)	E (90%–100% of VS)
(A)	Delay (s) (mean ± sd)				
0.3	−0.15 ± 13.7	−1.59 ± 11.9	−0.39 ± 11.2	0.207 ± 8.6	0.12 ± 6.1
0.4	0.95 ± 11.6	0.23 ± 9.3	0.949 ± 8.8	0.58 ± 6.2	0.17 ± 4.0
0.5	1.32 ± 8.4	0.53 ± 5.7	0.52 ± 5.0	0.44 ± 3.6	0.34 ± 3.1
(B)	Maximum correlation coefficient (mean ± sd)				
0.3	0.19 ± 0.3	0.22 ± 0.3	0.22 ± 0.3	0.26 ± 0.2	0.28 ± 0.2
0.4	0.27 ± 0.3	0.31 ± 0.3	0.32 ± 0.2	0.35 ± 0.2	0.37 ± 0.1
0.5	0.38 ± 0.3	0.44 ± 0.2	0.42 ± 0.2	0.45 ± 0.1	0.45 ± 0.1
(C)	CCF Precision				
0.3	0.13	0.12	0.10	0.09	0.08
0.4	0.12	0.11	0.10	0.09	0.08
0.5	0.12	0.10	0.09	0.07	0.06

**Table 3:** Statistics of MDS and VS percentages estimated on acquired EEG data.

Subject	Free Breathing Task			Breath Hold Task		
	MDS Properties			MDS Properties		
	N°	Length (s) (mean ± sd)	% of VS	N°	Length (s) (mean ± sd)	% of VS
1	9	10.08 ± 6.6	82.25	14	11.07 ± 8.0	67.41
2	2	16.63 ± 8.6	90.72	8	14.73 ± 8.7	66.70
3	6	10.79 ± 4.3	81.13	5	11.75 ± 2.8	89.79
4	16	11.02 ± 4.2	54.06	3	6.78 ± 0.4	94.44
5	7	15.62 ± 9.7	69.55	12	10.32 ± 2.4	65.73
6	11	11.17 ± 5.5	66.79	12	11.62 ± 5.8	61.39

**Table 4:** Correlation analysis results at single subject level between GFP estimated in delta band and P_ET_CO_2_. The time shfit with maximum correlation coefficient (Ts), the corresponding correlation coefficient (CC), and the critical values corresponding to α = 0.05 (Thr.).

Subject	Free Breathing Task			Breath Hold Task		
	Ts (s)	CC	Thr.	Ts (s)	CC	Thr.
1	−5	0.20	0.31	13	0.68 **	0.48
2	2	−0.26 *	−0.24	4	0.35 **	0.27
3	−5	0.37 *	0.37	1	0.22 **	0.15
4	−18	0.47 **	0.29	3	0.52 **	0.37
5	−9	0.34	0.38	−12	−0.73 **	−0.49
6	29	−0.26 *	−0.25	8	0.32	0. 46

* *p* < 0.05; ** *p* < 0.01.

**Table 5:** Maximum correlation coefficients and corresponding time shifts estimated at group level for simulated data. Both the simple and the weighted average results are shown.

	Target Values		Simple Average		Weighted Average	
	Delay (s)	Correlation Coefficient	Delay (s)	Correlation Coefficient	Delay (s)	Correlation Coefficient
			Mean ± sd	Mean ± sd	Mean ± sd	Mean ± sd
(a)	0	0.5	0.07 ± 0.3	0.46 ± 0.02	0.05 ± 0.3	0.46 ± 0.02
(b)	0	0.5	0.13 ± 0.5	0.46 ± 0.03	0.11 ± 0.4	0.46 ± 0.02
(c)	0	0.7	0.00 ± 0.01	0.70 ± 0.01	0.00 ± 0.01	0.70 ± 0.01
(d)	0	0.5	0.25 ± 0.9	0.46 ± 0.04	0.18 ± 0.6	0.46 ± 0.03

**Table 6:** Correlation analysis results at group level between GFP estimated in delta band and P_ET_CO_2_. The time shift with maximum correlation coefficient (Ts), the corresponding correlation coefficient (CC), and the critical values (Thr.) corresponding to α = 0.05 are shown.

	**Group Analysis-Simple Average**		
Task	Ts (s)	CC	Thr.
FB	−7	0.16 *	0.13
BH	6	0.34 *	0.16
	**Group analysis-Weighted Average**		
Task	Ts (s)	CC	Thr.
FB	−6	0.14 *	0.10
BH	5	0.35 *	0.15

* *p* < 0.05.

## Appendix A

Surrogate signals in case of MDS and Monte Carlo test of significance.

For two signals *x* and *y*, the cross-correlation function is calculated for each time lag ($r_{xy}\left(\tau \right)$). Under the null hypothesis (H_0_) of no correlation between signals, a Monte Carlo test is used to establish the significance of $r_{xy}\left(\tau \right)$. Specifically, from *x* and *y*, *N* uncorrelated surrogate signals $x_{i}$ and $y_{i}$ (with i=1,…,N) are calculated. These surrogate signals have these particular characteristics:

- For the amplitude spectra: if no Missing Data Segments (MDS) are present, all surrogate data have the same amplitude spectra of original data (|X(f)|,|Y(f)|). When in *x* and *y* signals MDS are present, the AutoCorrelation Functions (ACFs) of *x* and *y* must be estimated using only the sample available in each signal; the amplitude spectra are estimated from the square roots of the Fourier Transform of ACFs;
- The phase spectra ($\varphi_{x,i}\left(f\right),\varphi_{y,i}\left(f\right)$) are derived from random values between ±π with independent values and uniform distribution.

From the surrogate signals the cross-correlation functions are calculated ($r_{xy}\left(\tau \right)$) and the distribution of all coefficients approximates the sampling distribution for $r_{xy}\left(\tau \right)$, under H_0_. If $r_{xy}\left(\tau \right)$ lies outside the range of surrogate values (which represents the p-values and the statistical significance α), H_0_ is rejected.

## Appendix B

Normalized Root Mean Square Error

The Root Mean Square Error (RMSE) between a predicted time series $\hat{x}\left(t\right)$ and the actual time series x(t) is defined as

(B1) $$\text{RMSE}=\sqrt{\frac{\sum_{i=1}^{N}{\left(\hat{x}(i)-x(i)\right)}^{2}}{N}},$$

where *N* is the time series length.

We define the Normalized RMSE (NRMSE) as the RMSE normalized by the range of the predicted time series as

(B2) $$\text{NRMSE}=\frac{\text{RMSE}}{x_{\text{max}}-x_{\text{min}}},$$

where $x_{\text{max}}$ and $x_{\text{min}}$ are the maximum and minimum values of the predicted time series.

## Appendix C

Bayes Rule

The Bayes rule states that the posterior probability of the parameter θ given the data y can be written as

(C1) $$p\left(\theta |y\right)=\frac{p\left(y|\theta \right)p\left(\theta \right)}{p\left(y\right)},$$

where p(y) is constant with respect to θ, p(y|θ) is the likelihood of the data, and p(θ) are the priors about the parameter. In our case the priors are flat.
